# Serologic Evidence of Pandemic (H1N1) 2009 Infection in Dogs, Italy

**DOI:** 10.3201/eid1612.100514

**Published:** 2010-12

**Authors:** William G. Dundon, Paola De Benedictis, Elisabetta Viale, Ilaria Capua

**Affiliations:** Author affiliations: Istituto Zooprofilattico Sperimentale delle Venezie, Legnaro, Italy

**Keywords:** pandemic (H1N1) 2009, influenza A virus, viruses, dogs, Italy, letter

**To the Editor:** Until recently, the general consensus has been that dogs are poorly susceptible to natural infection with influenza A viruses; however, since the recent upsurge of influenza A circulating subtypes H5N1 and H1N1 viruses, cases of natural infection in dogs have apparently increased. Thus, the role of these animals is being reconsidered in the transmission and spread of influenza viruses (*1**–**3*).

In April 2009, the most recent of the human influenza A pandemics, pandemic (H1N1) 2009, was detected in Mexico. The virus rapidly spread worldwide, within weeks of its first isolation. To date, pandemic (H1N1) 2009 has primarily infected humans, although transmission from infected humans to other animals, including pigs, turkeys, ferrets, cats, and dogs has been reported (*4**,**5*).

In Italy (population ≈58 million), the first human cases of pandemic (H1N1) 2009 were reported in May 2009; confirmed cases peaked during the second week of November 2009 (week 46) (*6*). As of May 9, 2010, Italy had recorded an estimated 5,582,000 cases of pandemic (H1N1) 2009. In Italy as well, the population has ≈7 million companion dogs and ≈7.5 million cats (*7*). Because of the close contact between persons and their companion animals, we initiated this serologic study to determine whether evidence of pandemic (H1N1) 2009 transmission could be found in companion animals in Italy.

We tested serum specimens from dogs (n = 964) and cats (n = 97), originally submitted to the Istituto Zooprofilattico Sperimentale delle Venezie in Legnaro, Italy, from October through December 2009 (weeks 41–53), for assessment of rabies vaccine efficacy. An average of 70 samples were tested per week; the highest number of samples (n = 106) was tested for week 51 and the lowest (n = 25) for week 53. Testing for antibody to influenza A nucleoprotein was performed by using a commercially available competitive ELISA (cELISA) (ID Screen Influenza A Antibody Competition Assay; ID Vet, Montpellier, France), according to the manufacturer’s instructions. Previous work from our laboratory has assigned a sensitivity of 93.98% and specificity of 98.71% to this cELISA for the testing of canine serum samples (*8*). In total, 29 serum specimens tested at a 1:10 dilution, all from dogs, were positive after a second confirmatory screening. None of the 97 feline serum samples were positive by cELISA.

The cELISA-positive serum specimens were then treated with receptor-destroying enzyme (RDE; Sigma-Aldrich, St. Louis, MO, USA) (1 part serum: 3 parts RDE) for 16 h at 37°C, followed by heat inactivation at 56°C for 30 min. We then tested the specimens by the hemagglutination inhibition (HI) test against the pandemic virus A/Verona/Italy/2810/2009 (H1N1), A/swine/Italy/711/2006 (H1N1), and H3N8 (A/canine/Florida/2004) by using 0.5% chicken erythrocytes and standard methods (*9*). Seven serum samples (nos. 4410, 4438, 4444, 4460, 4517, 4520, 4681) were positive by HI to A/Verona/Italy/2810/2009 (H1N1) with titers ranging from 16 to 256 (Table), but not for the other viruses, although the samples with the higher titers of 256 (nos. 4460 and 4681) against A/Verona/Italy/2810/2009 also cross-reacted with antigen A/swine/Italy/711/2006 (H1N1) (titers 16 and 32, respectively).

**Table:** Anagraphic data and HI and MN titers of canine serum specimens against A/Verona/Italy/2810/2009 (H1N1), 2009*

Sample no.	Species	Birth date	Sample origin, Italy	Coordinates	Sample date/wk	HI	MN
4410	English setter	2005 Jun 5	Mercato Saraceno	43°57′0′′N,12°12′0′′E	Nov 24/43	32	640
4438	Labrador retriever	2005 Oct10	Casale Monferrato	45°8′3′′N, 8°27′30′′E	Nov 26/44	32	160
4444	Chihuahua	2008 Apr 7	Mantaova	45°10′0′′N, 10°48′0′′E	Nov 30/44	16	160
4460	Small cross-breed	2005 Jan 20	Milano	45°27′50.98′′N, 9°11′25.21′′E	Nov 30/44	256	>2,560
4517	Italian Segugio	2007 Jul 4	Giussano	45°42′0′′N, 9°13′0′′E	Nov 30/44	128	640
4520	Shih tzu	2007 Dec 8	Peschiera del Garda	45°26′0′′N, 10°41′0′′E	Dec 2/46	64	320
4618	Yorkshire terrier	2008 Dec 21	Garda	45°34′0′′N, 10°43′0′′E	Dec 9/45	256	>2,560

*HI, hemagglutination inhibition; MN, microneutralization.

The HI-positive serum specimens were later tested in a microneutralization assay with A/Verona/Italy/2810/2009 (H1N1). Suppression of virus antigen expression was assessed by an ELISA assay as endpoint by using a slight modification of a previously described procedure (*10*). As can be seen from the Table, all 7 serum specimens positive by HI for A/Verona/Italy/2810/09 inhibited infection of MDCK cells by the same virus at dilutions of 1:160 or higher, confirming the presence of anti-H1 antibodies.

To summarize, in this study, 1,061 serum specimens from companion dogs and cats collected during the circulation peak of pandemic (H1N1) 2009 in Italy were screened with 7 (0.7%) of the canine serum specimens showing evidence of exposure to the virus. Notably, the positive samples were collected during the period (weeks 43–45) that almost coincided with the reported peak for human cases of pandemic (H1N1) 2009 in Italy (week 46). Totals of 69, 77, and 56 samples were collected during weeks 43, 44, and 45, respectively, which indicates that the sample group had no bias.

The data thus suggest that transmission occurred, most probably by aerosol or close contact, between pandemic (H1N1) 2009–infected owners and their pets during this peak period of mid-November 2009. How long these animals were infected, whether the infection had clinical manifestations, and whether the dogs were capable of transmitting the virus to other hosts are all questions that remain unanswered. However, on the basis of the low number of positive specimens identified in this study, it would be unrealistic to suggest that dogs are particularly susceptible to pandemic (H1N1) 2009. Nevertheless, as has been seen with infection of dogs with subtype H3N8, influenza A viruses are quite capable of evolving and becoming more host specific. This factor alone would justify the continued surveillance of influenza A viruses in domestic dogs.
